# Loss of Serine-Type D-Ala-D-Ala Carboxypeptidase DacA Enhances Prodigiosin Production in *Serratia marcescens*

**DOI:** 10.3389/fbioe.2019.00367

**Published:** 2019-12-03

**Authors:** Xuewei Pan, Changhao Sun, Mi Tang, Chao Liu, Jianing Zhang, Jiajia You, Tolbert Osire, Yang Sun, Youxi Zhao, Meijuan Xu, Taowei Yang, Zhiming Rao

**Affiliations:** ^1^Key Laboratory of Industrial Biotechnology of the Ministry of Education, Laboratory of Applied Microorganisms and Metabolic Engineering, School of Biotechnology, Jiangnan University, Wuxi, China; ^2^Biochemical Engineering College, Beijing Union University, Beijing, China

**Keywords:** D-Ala-Ala carboxypeptidase DacA, prodigiosin synthesis, prodigiosin leakage, feedback inhibition, *Serratia marcescens*

## Abstract

*Serratia marcescens*, a gram-negative bacterium, found in a wide range of ecological niches can produce several high-value products, including prodigiosin, althiomycin, and serratamolide. Among them, prodigiosin has attracted attention due to its immunosuppressive, antimicrobial, and anticancer properties. However, the regulatory mechanisms behind prodigiosin synthesis in *Serratia marcescens* remains limited. Here, a transposon mutant library was constructed to identify the genes related to prodigiosin synthesis, and *BVG90_02415* gene encoding a peptidoglycan synthesizing enzyme D-Ala-D-Ala carboxypeptidase DacA was found to negatively regulates prodigiosin synthesis. Quantitative measurements revealed that disruption of *dacA* increased prodigiosin production 1.46-fold that of the wild-type strain JNB5-1 in fermentation medium. By comparing differences in cell growth, *pigA* gene expression level, cell morphology, membrane permeability, and intracellular prodigiosin concentration between wild-type strain JNB5-1 and *dacA* mutant SK4-72, results revealed that the mechanism for hyper-producing of prodigiosin by the *dacA* mutant was probably that *dacA* disruption enhanced prodigiosin leakage, which in turn alleviated feedback inhibition of prodigiosin and increased expression of *pig* gene cluster. Collectively, this work provides a novel insight into regulatory mechanisms of prodigiosin synthesis and uncovers new roles of DacA protein in regulating cell growth, cell morphology, and membrane permeability in *Serratia marcescens*. Finally, this study offers a new strategy for improving production of high-value compounds in *Serratia marcescens*.

## Introduction

*Serratia marcescens* (*S. marcescens*), is a gram-negative rod-shaped bacterium of the Enterobacteriaceae family found in a wide range of environments like soil, water, plants, insects, foods, and machinery (Abreo and Altier, 2019). Studies on *S. marcescens* have shown that it can produce many high-value metabolites and enzymes, such as prodigiosin (Yip et al., 2019), biosurfactant (Matsuyama et al., 1992), oocydin A (Matilla et al., 2017), althiomycin (Gerc et al., 2012), bacteriocins (Foulds, 1972), serratin (Luna et al., 2013), serratiopeptidase (Velez-Gomez et al., 2019), chitinase (Emruzi et al., 2018), and fibrinolytic serine metalloprotease (Krishnamurthy and Belur, 2018). Among them, prodigiosin, a red linear tripyrrole pigment (Figure S1), has attracted attention owing to its antimalarial, antibacterial, antifungal, antiprotozoal, and immunosuppressant activities (Clements et al., 2019). Many studies have been used to dissect its biosynthetic pathways to increase the production of prodigiosin as it has the potential to become a clinical drug.

Unfortunately, although nearly 30 genes have been reported to be involved in the biosynthesis of prodigiosin in *S. marcescens*, the understanding of the regulatory network for the prodigiosin synthesis in *S. marcescens* is still limited. All identified genes related to prodigiosin synthesis in *S. marcescens* could be divided into two groups, the first group involves genes in the biosynthetic pathway of prodigiosin synthesis and the other group includes genes encoding transcriptional regulators. The prodigiosin biosynthesis pathway consists of a total of 14 genes, named *pigA, pigB, pigC, pigD, pigE, pigF, pigG, pigH, pigI, pigJ, pigK, pigL, pigM*, and *pigN*, whereby the *pigB, pigD*, and *pigE* genes are responsible for the synthesis of monopyrrole moiety (MAP), while the *pigA, pigF, pigG, pigH, pigI, pigJ, pigM*, and *pigN* genes are responsible for the synthesis of bipyrrole moiety (MBC). Finally, the *pigC* gene encodes the terminal condensing enzyme PigC that condenses MAP and MBC to prodigiosin (Williamson et al., 2006a). In *S. marcescens*, the synthesis of prodigiosin is also controlled by several transcriptional regulators, including positive regulators EepR (Shanks et al., 2017), PigP (Shanks et al., 2013), SmaI (Coulthurst et al., 2006), GumB (Stella et al., 2018), and RbsR (Lee et al., 2017), and negative regulators CopA (Williamson et al., 2006b), CRP (Stella and Shanks, 2014), HexS (Stella et al., 2012), RssB (Horng et al., 2010), and SpnR (Horng et al., 2002). Among them, HexS, EepR, PigP, and RssB regulators directly bind to the promoter regions of the prodigiosin-associated *pig* operon, and thus affect the synthesis of prodigiosin. On the other hand, CRP directly binds to the promoter region of the *eepR* gene, and negatively regulates the synthesis of prodigiosin.

*S. marcescens* JNB5-1 was isolated from soil samples and it produced a relatively high amount of prodigiosin (6.35 g/L) when fermented in fermentation medium. However, the molecular mechanism behind prodigiosin synthesis in this strain was still limited. In this study, a Tn*5G* transposon insertion mutant library was constructed using *S. marcescens* JNB5-1 as the parental strain, and serine-type D-Ala-D-Ala carboxypeptidase DacA was identified to negatively regulates prodigiosin synthesis in *S. marcescens*. By analyzing the effect of DacA on cell growth, cell morphology, *pigA* gene expression level, permeability of inner and outer membranes, and intracellular prodigiosin concentration, the results revealed that the probable mechanism behind DacA affecting the prodigiosin biosynthesis. Furthermore, the role of the other three serine-type D-Ala-D-Ala carboxypeptidase DacB, DacC, and DacD on prodigiosin synthesis was also analyzed.

## Materials and Methods

### Bacterial Strains and Growth Conditions

*S. marcescens* JNB5-1 is a prodigiosin producing strain collected in our laboratory. *E. coli* DH5α and *E. coli* S17-1 λpair were used for plasmid construction. The *E. coli* strains were grown in LB medium at 37°C, and the *S. marcescens* strains were grown in LB medium or fermentation medium at 30°C. When appropriate, the medium was supplemented with kanamycin (50 μg/mL), ampicillin (50 μg/mL), apramycin (50 μg/mL), or gentamicin (10 μg/mL) for *E. coli* strains cultivation; and apramycin (50 μg/mL), kanamycin (150 μg/mL), clindamycin (50 μg/mL), or gentamicin (50 μg/mL) for *S. marcescens* strains cultivation. The bacterial strains, and plasmids used in this study are listed in Table 1.

**Table 1:** Strains and plasmids used in this study.

**Strains and plasmids**	**Description**	**Source**
***E. coli*** **strains**		
DH5α	*hsdR recA lacZYAF80 lacZDM15*	BRL
S17-1 (λpir)	F^−^*recA hsdR* RP_4−2_ (Tc::Mu)(Km::Tn7) lysogenized with λpir phage	Laboratory collection
S17-1/pXW1804-dacB	*E. coli* S17-1 containing pXW1804-dacB plasmid	This Study
S17-1/pXW1805-dacC	*E. coli* S17-1 containing pXW1805-dacC plasmid	This Study
S17-1/pXW1806-dacD	*E. coli* S17-1 containing pXW1806-dacD plasmid	This Study
***S. marcescen*****s strains**		
JNB5-1	*S. marcescens* wild type strain	Laboratory collection
SK4-72	*dacA*::Gm^R^ mutant of JNB5-1, prodigiosin hyper-producing mutant	This Study
SK4-72/pXW1803	Mutant SK4-72 with pXW1803 plasmid	This Study
JNB5-1ΔdacB	*S. marcescens* JNB5-1 *dacB*^−^ mutant, Apr^R^	This Study
JNB5-1ΔdacC	*S. marcescens* JNB5-1 *dacC*^−^ mutant, Apr^R^	This Study
JNB5-1ΔdacD	*S. marcescens* JNB5-1 *dacD*^−^ mutant, Apr^R^	This Study
**Plasmids**		
pRK2013Tn*5G*	Tn*5G* carrying plasmid, Km^R^Gm^R^	Nunn and Lory, 1992
pMD18T	Cloning vector, 2,692 bp, Amp^R^, lacZ	TaKaRa
pACYC177	low copy vector plasmid, Amp^R^Km^R^	Laboratory collection
pXW1803	*dacA* gene with its own promoter cloned in pACYC177, Amp^R^Km^R^	This Study
pUTKm	Tn5-based delivery plasmid with Km^R^Amp^R^	Herrero et al., 1990
pXW1804-dacB	Recombinant plasmid used for *dacB* gene knockout, Apr^R^Cli^R^	This Study
pXW1805-dacC	Recombinant plasmid used for *dacC* gene knockout, Apr^R^Cli^R^	This Study
pXW1806-dacD	Recombinant plasmid used for *dacD* gene knockout, Apr^R^Cli^R^	This Study

### Screening of Prodigiosin Producing Mutants

Tn*5G* transposon was used to mutagenize *S. marcescens* JNB5-1 in order to identify prodigiosin producing mutants as previously described (Nunn and Lory, 1992). Briefly, *E. coli*/pRK2013 Tn*5G* was used as the donor strain and *S. marcescens* JNB5-1 was used as the recipient strain. After mating, the mutant library was plated onto L-agar medium containing gentamicin (50 μg/mL) and ampicillin (50 μg/mL). Then, the transposon mutants were selected according to the color intensity of the colonies before fermentation in 48-well plates in LB medium to determine prodigiosin production. Finally, high and low prodigiosin producing mutants were isolated.

### Prodigiosin Production Assay in Shake Flask Fermentation

The ability of JNB5-1, SK4-72, and SK4-72/pXW1803 strains to produce prodigiosin was determined by shake-flask fermentation in LB medium or fermentation medium (Sucrose 2%, Beef extract 1.5%, CaCl_2_ 1%, L-proline 0.75%, MgSO_4_·7H_2_O 0.02%, and FeSO_4_·7H_2_O 0.006%). The method to determine prodigiosin yield was by acidified ethanol and absorbance measured (A_535_), as previously described (Kalivoda et al., 2010). The amount of prodigiosin produced by different strains was calculated according to the standard curve: Y = 1.1936X – 0.001 (Y indicates that the fermentation broth soluble in acid ethanol at pH 3.0, and the wavelength measured at A_535_. X indicates the amount of prodigiosin produced by strains).

### Identification of the Transposon Insertion Sites and Complementation Experiment

To identify the insertional sites of Tn*5G* transposon in prodigiosin producing mutants, inverse PCR was performed as previously described (Wang et al., 1996). Briefly, genomic DNA of mutants were isolated, digested by the restriction enzyme *Taq*I, self-ligated, and amplified using primers OTn1 and OTn2 showed in Table 2. The PCR products were then cloned into pMD18-T vector (TaKaRa) for sequencing and the obtained sequences were analyzed by searching the database GenBank of NCBI (http://www.ncbi.nlm.nih.gov/). The primers DacA-F and DacA-R listed in Table 2 were designed to amplify the *dacA* gene with its native promoter and cloned into the pACYC177 plasmid. The recombinant plasmid was then transformed into the SK4-72 mutant for complementation test.

**Table 2:** Primers used in this study.

**Primer**	**Primer sequence (5^**′**^-3^**′**^)**	**Function**
OTn1	GATCCTGGAAAACGGGAAAG	Identification of Tn*5G* in prodigiosin production mutants
OTn2	CCATCTCATCAGAGGGTAGT	
RT-pigA-F	ATGGCTTTATGGGCGTGTCC	qPCR primers, coding region of *pigA* gene
RT-pigA-R	GAGAGGCAATCTTTCGGGCT	
RT-16S-F	CACACCGCCCGTCACACCA	qPCR primers, coding region of 16S rRNA gene
RT-16S-R	CGCAGGTTCCCCTACGGTTAC	
DacA-F	TTAACCGAACCAGTGGTGGAACATC	Amplification of *dacA* gene with its native promoter
DacA-R	TTAAGCCCGGCGGGCATTCA	
DacB-U-F	TAGGCCGAATTCGAGCTCGGTACCCGG ACAGGGTTTCGTCCACC	Amplification of *dacB* gene upstream homologous arms
DacB-U-R	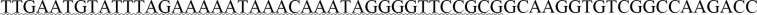	
DacB-D-F	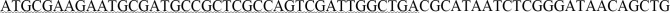	Amplification of *dacB* gene downstream homologous arms
DacB-D-R	ATTACAGCCGGATCCCCGGGTACCG TAAAAATCGCCGCTGAAGATCGC	
DacC-U-F	TAGGCCGAATTCGAGCTCGGTACCGAT GGCGTCCACGTCATGC	Amplification of *dacC* gene upstream homologous arms
DacC-U-R	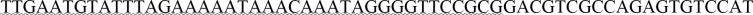	
DacC-D-F	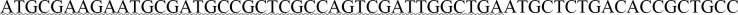	Amplification of *dacC* gene downstream homologous arms
DacC-D-R	ATTACAGCCGGATCCCCGGGTACCTCGACTTTGGGCAGATCCGC	
DacD-U-F	TAGGCCGAATTCGAGCTCGGTACCGTGTCGCCCACCAGCTTAGG	Amplification of *dacD* gene upstream homologous arms
DacD-U-R	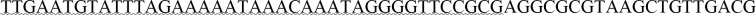	
DacD-D-F	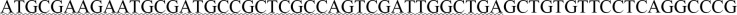	Amplification of *dacD* gene downstream homologous arms
DacD-D-R	ATTACAGCCGGATCCCCGGGTACCGGC GGCGTTTTTCAGGCTG	
Apr-D-F	CGCGGAACCCCTATTTGTTTATTTTTC	Amplification of *aacC3* gene
Apr-D-R	TCAGCCAATCGACTGGCGAGC	
DacB-F	CCTGCTTTTCCATCAGTTCCAGG	Primers used to confirm *dacB* gene knockout succeed
DacB-R	ATCAGTCCAAGTGGCCCATCTTC	
DacC-F	GCCGATGCGGTAAGCATTGC	Primers used to confirm *dacC* gene knockout succeed
DacC-R	ATCAGTCCAAGTGGCCCATCTTC	
DacD-F	GGCCTTTATTATCGGCCAAACGG	Primers used to confirm *dacD* gene knockout succeed
DacD-R	ATCAGTCCAAGTGGCCCATCTTC	

*The sequences underlined stand for the homologous sequence of the pUTKm plasmid*.

*The sequences wavelined stand for the homologous sequence of the apramycin resistance aacC3 gene*.

### Growth Assay

To analyze the growth of *S. marcescens* JNB5-1, SK4-72, and SK4-72/pXW1803, 3% volume of the exponential-phase cells (OD_600_ at 0.6) of these strains was inoculated into fresh LB medium. The growth of *S. marcescens* cells was determined by monitoring the optical density (OD_600_) of cultures at 0, 2, 4, 6, 8, 10, 12, 24, 36, 48, and 60 h time intervals, and the growth curves were plotted as the values of OD_600_ nm vs. the incubation time.

### Real-Time Quantitative PCR (RT-qPCR) Assay

*S. marcescens* JNB5-1 and SK4-72 were fermented in LB medium, and the samples were collected after 4, 6, 8, 10, 12, 24, 36, 48, and 60 h to analyze prodigiosin-synthesis related *pigA* gene expression levels in the two strains by RT-qPCR. The RNAprep pure Kit (TIANGEN) was used to extract total RNA of the collected cells, and the obtained total RNA (0.5 μg) was subjected to reverse transcription to synthesize complementary DNA (cDNA) using the HiScript® II Q RT SuperMix (Vazyme). Then, the cDNA was diluted to 200 ng/μL and subjected to real-time quantitative PCR (RT-qPCR) analysis using the ChamQTM Universal SYBR® qPCR Master Mix (Vazyme). The 16S ribosomal RNA protein encoding gene was used as an internal control. The primers RT-pigA-F/RT-pigA-R and RT-16S-F/RT-16S-R used for RT-qPCR analysis are listed in Table 2.

### Scanning Electron Microscopy

Scanning electron microscope (SEM) Hitachi SU8220 (Hitachi, Tokyo, Japan) was used to observe cell shapes of JNB5-1, SK4-72, and SK4-72/pXW1803 strains. Bacterial cells were cultured in LB medium for 12 h at 28°C with shaking at 200 rpm, and then 100 μL bacterial cells were plated onto LB-agar medium for 12 h at 28°C. Colonies of *S. marcescens* were re-suspended in sterile water and placed on a carbon film-coated copper grid (230 meshes; Beijing Zhongjing Science and Technology Co., Ltd., Beijing, China). Finally, the bacteria liquid on the film was dried at 25°C and observed by SEM. The software ImageJ was used to analyze the length and width of the cells (https://imagej.nih.gov/ij/download.html). Forty cells of each indicated strain were used for the analysis.

### Outer and Inner Membrane Permeability Determination

Outer membrane permeability of *S. marcescens* JNB5-1, SK4-72, and SK4-72/pXW1803 cells was evaluated as previously described (Yang et al., 2018). Firstly, 20 μL N-Phenyl-1-naphthylamine (NPN, 100 mM) was mixed with 200 μL logarithmic cell suspension (OD_600_ at 0.8). Then, the NPN fluorescence intensity of samples in black 96-well plate (Corning® 3603) was determined immediately by using Biotek Cytation 3 microplate reader. The excitation and emission wavelengths of Biotek Cytation 3 were 350 and 420 nm, respectively. Inner membrane permeability of *S. marcescens* JNB5-1, SK4-72, and SK4-72/pXW1803 cells were evaluated by measuring the absorbance of 2-Nitrophenyl β-D-galactopyranoside (ONPG) according to the previously described methods (Lehrer et al., 1988). Twenty microliters ONPG (100 mM) was mixed with 200 μL logarithmic cell suspension (OD_600_ at 0.8), and then incubated at 37°C for 65 min and sampled every 5 min. The absorbance of ONPG in 96-well plate (Corning, NY, USA) was measured by BioTek Epoch 2 microplate reader at the wavelength of 420 nm. NPN and ONPG were purchased from Shanghai Aladdin Bio-Chem Technology Co., Ltd.

### Determination of Intracellular and Extracellular Prodigiosin Concentrations

The intracellular and extracellular prodigiosin concentrations of wild-type strain JNB5-1, *dacA* mutant SK4-72, and complementary strain SK4-72/pXW1803 were determined at different fermentation time intervals (0, 12, 24, 36, 48, 60, and 72 h) in triplicate. After 1 mL fermentation broth of JNB5-1, SK4-72, and SK4-72/pXW1803 strains were harvested, and centrifuged at 12,000 rpm for 10 min, the supernatant and pellets were collected and resuspended in acidified ethanol, respectively prior to measurement of absorbance (A_535_). Then, the intracellular and extracellular prodigiosin concentrations of JNB5-1, SK4-72, and SK4-72/pXW1803 strains were calculated according to the standard curve Y = 1.1936X – 0.001 (Y indicated that the fermentation broth soluble in acid ethanol at pH 3.0, and the wavelength measured at A_535_. X indicated the amount of prodigiosin produced by strains).

### Construction of the *dacB, dacC*, and *dacD* Gene Deletion Strains

The method used for D-Ala-D-Ala carboxypeptidase encoding gene *dacB* knock out was shown in Figure S2. Firstly, using the published *S. marcescens* UMH8 genome sequence (CP018927.1), primers for *dacB* gene knock out were designed (Table 2), and the upstream and downstream homologous arms (DacB-up and DacB-down) of *dacB* gene with a length of about 1,000 bp was obtained by PCR using JNB5-1 genome as the template. Meanwhile, primers Apr-D-F and Apr-D-R were designed to amplify the apramycin resistance marker AacC3. Secondly, Using DNA fragments DacB-up, DacB-down, and Apr resistance marker AacC3 as templates, DacB-U-F and DacB-D-R as primers, with overlap extension PCR, the DacB-up-AacC3-DacB-down DNA fragment was obtained. Then, using ClonExpress II One Step Cloning Kit (Vazyme), the recombinant plasmid pXW1805/*dacB* was constructed by cloning the DacB-up-AacC3-DacB-down DNA fragment into the pUTKm plasmid, and the recombinant strain *E. coli* S17-1/pXW1804-dacB was obtained. Finally, using *E. coli* S17-1/pXW1804-dacB as donor strain and *S. marcescens* JNB5-1 as recipient strain, after mating, *dacB* gene deletion mutant were screened on the apramycin resistance plate. The genomic DNA of the *dacB* mutants was extracted and the absence of the *dacB* gene in mutant JNB5-1ΔDacB was confirmed by PCR and sequencing. In the same way, we also knocked out the *dacC* and *dacD* genes, and the mutant JNB5-1ΔDacC, and JNB5-1ΔDacD were obtained (Figure S2). Primers used for *dacB, dacC* and *dacD* genes knock out are shown in Table 2.

### Statistical Analysis

The experiments in this work were performed independently at least three times, and data are expressed as mean and standard deviation (SD). To compare statistical differences between groups of experimental data, student's *t*-test or one-way ANOVA with Tukey's post-test was used.

## Results

### Identification of Genes Related to Prodigiosin Synthesis in *S. marcescens*

**Figure 1:**
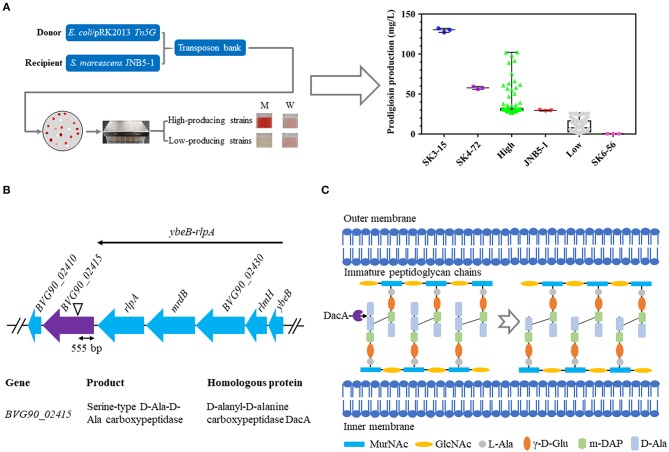
Construction of a random Tn*5G* transposon library to identify genes involved in prodigiosin synthesis. **(A)** Prodigiosin production assay of the mutants isolated from Tn*5G* transposon insertion mutation. JNB5-1 is parental strain used for screening prodigiosin-synthesis mutants (*n* = 1). SK4-72 and SK3-15 are prodigiosin high-producing mutants with *dacA* and *lrhA* genes disrupted, respectively (*n* = 2). SK6-56 is prodigiosin low-producing mutant with *pigC* gene disrupted (*n* = 1). High indicates prodigiosin high-producing mutants (*n* = 103). Low indicates prodigiosin low-producing mutants (*n* = 160). **(B)** The genetic loci identified in prodigiosin hyper-producing mutant SK4-72. Black triangle indicates the Tn*5G* transposon insertion site. *ybeB*-*rlpA* operon including gene *ybeB, rlmH, BVG90_02430, mrdB*, and *rlpA*. **(C)** Peptidoglycan chain synthesis in *S. marcescens*. The role of the D-Ala-D-Ala-carboxypeptidases DacA is delete the C-terminal D-Ala from the peptidoglycan precursor pentapeptide molecules. For **(A)**, the experiments were performed in biological triplicates. Error bars indicate the standard deviations.

To identify the genes involved in prodigiosin synthesis in *S. marcescens*, a random Tn*5G* transposon insertion library using *E. coli*/pRK2013 Tn*5G* as the donor strain and *S. marcescens* JNB5-1 as the recipient strain was constructed, and nearly 20,000 mutants were collected (Figure 1A). Further, based on the colored colonies on the screening plates, 266 mutants were selected and fermented in 48-well plates for 24 h in LB medium to determine the ability of mutants to synthesize prodigiosin. It was found that the color intensity of the colony positively correlated to the amount of prodigiosin produced, that is, the more red the colony, the higher concentration of prodigiosin produced by the strains. We finally isolated high and low prodigiosin producing mutants according to the prodigiosin production between SK6-56 and SK3-15 mutants (Figure 1A, Table S1).

Further, the transposon insertion sites of 28 low prodigiosin producing mutants and four high prodigiosin producing mutants were determined by inverse PCR to identify the genes involved in prodigiosin synthesis. For 28 low prodigiosin producing mutants, Tn*5G* was inserted into 19 different genes or intergenic regions (Table S1), and these sites fall into three categories: (i) genes with a known role in prodigiosin production, including *pigC* (Williamson et al., 2006a), *pigF* (Williamson et al., 2006a), and *rbsR* (Lee et al., 2017) genes. (ii) genes with unknown roles in prodigiosin synthesis, including adenylate cycle encoding gene *BVG90_22210*, sulfite reductase [NADPH] flavoprotein alpha-component encoding gene *BVG90_00600*, cupin encoding gene *BVG90_17190*, phage holin encoding gene *BVG90_14345*, transcriptional regulator encoding genes *BVG90_02010* and *BVG90_03255*, hypothetical protein encoding genes *BVG90_17590* and *BVG90_02925*, ubiquinone biosynthesis regulatory protein kinase UbiB encoding gene *BVG90_22540*, acetolactate synthase isozyme one small subunit encoding gene *BVG90_00235*, alkaline phosphatase encoding gene *BVG90_04215*, peptide synthetase encoding gene *BVG90_22885* and alcohol dehydrogenase encoding gene *BVG90_07405*. (iii) intergenic regions, including *BVG90_24610* and *BVG90_24615* genes intergenic region, *BVG90_13460* and *BVG90_13465* genes intergenic region and *BVG90_18410* and *BVG90_18145* genes intergenic region (Table S1).

For four prodigiosin high-yielding mutants, Tn*5G* was inserted into four different sites, including: (i) genes encoding a negative regulator which has been reported to be involved in prodigiosin biosynthesis, including carbon storage regulator RsmA (*rsmA*) (Hampton et al., 2016) and transcriptional regulator HexS (*hexS*) (Tanikawa et al., 2006); (ii) gene encoding transcriptional regulator MetR; (iii) D-Ala-D-Ala carboxypeptidase DacA encoding gene *BVG90_02415* (Table S1). The goal of this study was to determine whether D-Ala-D-Ala carboxypeptidase DacA affected prodigiosin synthesis in *S. marcescens* and the molecular mechanism behind its regulation. And the role of other important genes for prodigiosin synthesis will be described elsewhere.

### D-Ala-D-Ala Carboxypeptidase DacA Negatively Regulates Prodigiosin Synthesis in *S. marcescens*

SK4-72, a hyper-prodigiosin producing mutant, was identified by the method described above, and the prodigiosin yield of this mutant was 1.96 times that of wild-type strain JNB5-1 (29.34 mg/L) when fermented in 48-well plate for 24 h in LB medium (Figure 1A, Table S1). The disrupted gene of SK4-72 mutant was identified by inverse PCR analysis, and Tn*5G* was found inserted between 555 and 556 bp in the coding region of *BVG90_02415* (*dacA*) gene (Figure 1B, Table S1). The *dacA* gene encodes a D-Ala-D-Ala carboxypeptidase DacA, which specifically cleaves to the D-Ala→D-Ala bond in pentapeptides, leading to the release of the terminal D-Ala and affecting the maturation of the cell wall peptidoglycan (Figure 1C).

**Figure 2:**
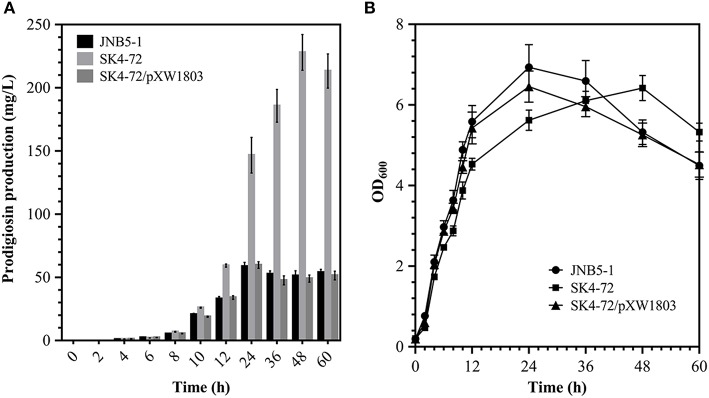
Effect of DacA on prodigiosin production and cell growth. **(A)** Prodigiosin production was significantly increased in *dacA* mutant SK4-72. **(B)** Growth curves of JNB5-1, SK4-72, and SK4-72/pXW1803 strains in LB medium. JNB5-1 is wild-type strain, SK4-72 is *dacA* mutant, and SK4-72/pXW1803 is *dacA*-complemented strain. Plasmid pXW1803 carries gene *dacA* with its own promoter. The experiments were performed in biological triplicates. Error bars indicate the standard deviations.

Further, to analyze the effect of DacA on prodigiosin biosynthesis, we determined the ability of wild-type strain JNB5-1 and *dacA* mutant SK4-72 to produce prodigiosin by shake flask fermentation in LB medium. As shown in Figure 2A, wild-type strain JNB5-1 produced utmost 58.67 mg/L of prodigiosin, while *dacA* mutant SK4-72 produced 146.63 mg/L prodigiosin after 24 h of fermentation, which was 2.50 times of wild-type strain JNB5-1. Importantly, unlike the wild-type strain JNB5-1, there was continued increase in prodigiosin production in the *dacA* mutant SK4-72 after 24 h of fermentation, and reached a highest production of 227.96 mg/L at 48 h, which was 3.89 times that of wild-type strain JNB5-1 (Figure 2A). With the complementary experiment, the *dacA* gene was further confirmed to be associated with prodigiosin synthesis (Figure 2A). These results indicated that D-Ala-D-Ala carboxypeptidase DacA significantly affected the biosynthesis of prodigiosin in *S. marcescens*.

Further, to reveal whether cell-growth caused higher production of prodigiosin in the *dacA* mutant SK4-72 compared to wild-type strain JNB5-1, growth of wild-type strain JNB5-1, *dacA* mutant SK4-72, and complementary strain SK4-72/pXW1803 were determined. After disruption of *dacA* gene, the growth rate of *S. marcescens* cells was significantly inhibited (Figure 2B), suggesting that DacA was necessary for the growth of *S. marcescens* and DacA possibly affected the synthesis of prodigiosin by influencing the expression level of prodigiosin-associated *pig* gene cluster at transcription level.

### Effect of *dacA* Disruption on Prodigiosin-Associated *pig* Operon Expression

**Figure 3:**
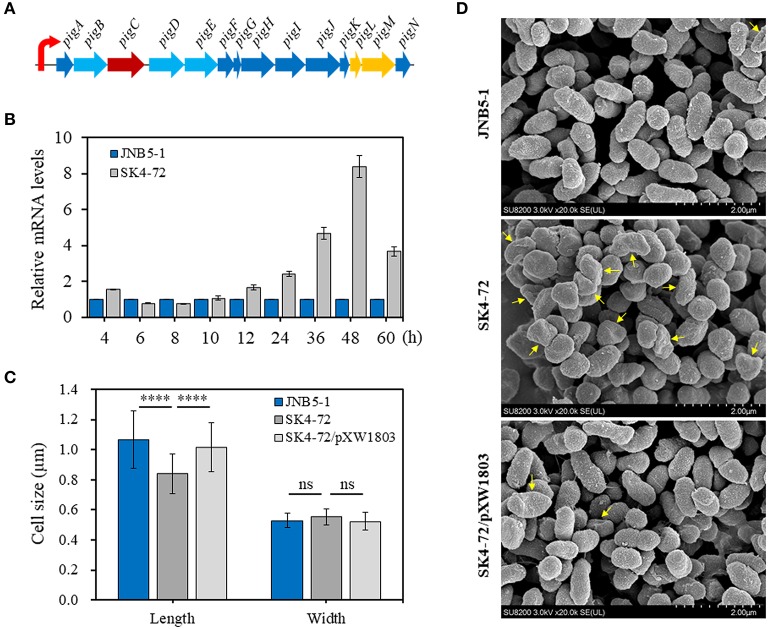
Influence of DacA on prodigiosin-synthesis gene *pigA* expression and cell shape. **(A)** Gene organization of the prodigiosin biosynthetic gene clusters in *S. marcescens*. These 14 genes were transcribed as a polycistronic mRNA from a promoter upstream of *pigA* gene. **(B)** RT-qPCR analysis of the *pigA* gene expression level in the strains JNB5-1 and *dacA* mutant SK4-72. **(C)** SEM images of the cells of JNB5-1, SK4-72, and SK4-72/pXW1803 strains. **(D)** Measurements of cell length and width of the indicated cells (40 cells for each strains). For **(B)**, the experiment was performed in biological triplicates. Error bars indicate the standard deviations. For **(D)**, one-way ANOVA was used to examine the mean differences between the data groups. ****p < 0.0001; ns, No significant difference.

The metabolic pathway of prodigiosin synthesis in *S. marcescens* was regulated by *pig* gene cluster, a total of 14 genes. These 14 genes are located on the same operon and under control of a promoter upstream of *pigA* gene (Figure 3A). To reveal the cause of hyper-production of prodigiosin in the *dacA* mutant SK4-72, the relative expression levels of *pigA* gene, the first gene in *pig* operon, were determined by RT-qPCR at different fermentation time intervals in the *dacA* mutant SK4-72 and wild-type strain JNB5-1. As shown in Figure 3B, after 4 h fermentation, the expression level of *pigA* gene in *dacA* mutant SK4-72 was 1.56-times of wild-type strain JNB5-1. After 6–10 h fermentation, there was no significant difference in the expression level of *pigA* in the *dacA* mutant SK4-72 and JNB5-1. However, the expression levels of *pigA* gene in *dacA* mutant SK4-72 were 1.66-, 2.41-, 4.67-, 8.40-, and 3.67-times that of wild-type strain JNB5-1, after 12, 24, 36, 48, and 60 h fermentation, respectively (Figure 3B). These results suggested that the higher production of prodigiosin in the *dacA* mutant SK4-72 could be correlated with the higher expression levels of the prodigiosin-associated *pig* operon.

### DacA Is Required to Maintain Normal Cell Morphology

D-Ala-D-Ala-carboxypeptidase DacA, low molecular weight penicillin-binding proteins (PBPs), play important roles in affecting the synthesis of bacterial cell walls and maintaining normal cell morphology by mediating peptidoglycan cross-linking, structural stability and cell wall modification (Yang et al., 2019). The cell morphology of *dacA* mutant SK4-72, wild-type strain JNB5-1 and complementary strain SK4-72/pXW1803 were observed by SEM. Measuring the length and width of 40 cells of these three strains, showed that the disruption of *dacA* resulted in a significant decrease in cell length, the average length for wild-type strain was 1.06 mm, and only 0.84 mm for the *dacA* mutant SK4-72 (Figures 3C,D, *P* < 0.001); and a slight increase in bacterial width, with the average width for wild-type strain is 0.53 and 0.55 mm for *dacA* mutant SK4-72 (Figures 3C,D). Moreover, we also observed that when *dacA* was disrupted, the strain produced more irregular cells (Figure 3D, yellow arrow). These results indicated that *dacA* was involved in regulation of cell morphology, and thus its absence probably affected the permeability of the cell membrane in *S. marcescens*.

### DacA Regulates Cell Membrane Permeability

**Figure 4:**
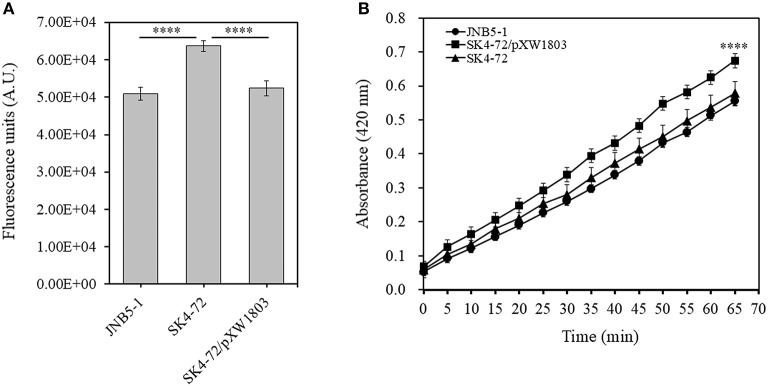
DacA negatively regulates permeability of the outer and inner membranes. **(A)** Effects of *dacA* disruption on outer membrane permeability. **(B)** Effects of *dacA* disruption on inner membrane permeability. JNB5-1 is wild-type strain, SK4-72 is *dacA* mutant, and SK4-72/pXW1803 is *dacA*-complemented strain. The experiments were performed in biological triplicates. Error bars indicate the standard deviations. One-way ANOVA was used to examine the mean differences between the data groups. ****p < 0.001.

N-Phenyl-1-naphthylamine (NPN) and 2-Nitrophenyl β-D-galactopyranoside (ONPG) have been used as probes to evaluate the permeability of the outer and inner membranes previously (Lehrer et al., 1988; Yang et al., 2018). To verify whether *dacA* affects cell membrane permeability in *S. marcescens*, NPN and ONPG were used to analyze the cell membrane permeability of JNB5-1, SK4-72, and SK4-72/pXW1803 strains. The fluorescence intensity of NPN in aqueous environment is weak, but could increase in hydrophobic or non-polar environments. When the network of peptidoglycan was disrupted leading to the formation of incomplete cell wall, the permeability of the outer membrane is enhanced, resulting in the increased cell binding capacity of NPN and NPN fluorescence intensity. As shown in Figure 4A, compared with the wild-type strain JNB5-1 (50,884.3 A.U.) and complementary strain SK4-72/pXW1803 (52,390.2 A.U.), the NPN fluorescence intensity of *dacA* mutant SK4-72 significantly increased (63,680.0 A.U., *P* < 0.001), indicating that the absence of *dacA* in *S. marcescens* probably affected the permeability of outer membrane.

Compared with the intact cells, when the permeability of inner membrane was enhanced, the intracellular β-galactosidase quickly hydrolyzed ONPG. Therefore, the ONPG hydrolysis ability of strains JNB5-1, SK4-72, and SK4-72/pXW1803 was analyzed. Compared with strains JNB5-1 and SK4-72/pXW1803, the ONPG hydrolysis ability of *dacA* mutant SK4-72 was significantly enhanced, suggesting that *dacA* disruption enhanced the permeability of inner membrane of *S. marcescens* (Figure 4B, *P* < 0.001). Taken together, we speculated that the cause for hyper-production of prodigiosin in the *dacA* mutant SK4-72 was due to enhanced cell membrane permeability as a result of *dacA* disruption and this facilitated prodigiosin leakage, which in turn alleviated the feedback inhibition of prodigiosin, and improved the expression level of the *pig* operon. Finally, the yield of prodigiosin increased significantly.

### Effect of DacA on Prodigiosin Production in Fermentation

**Figure 5:**
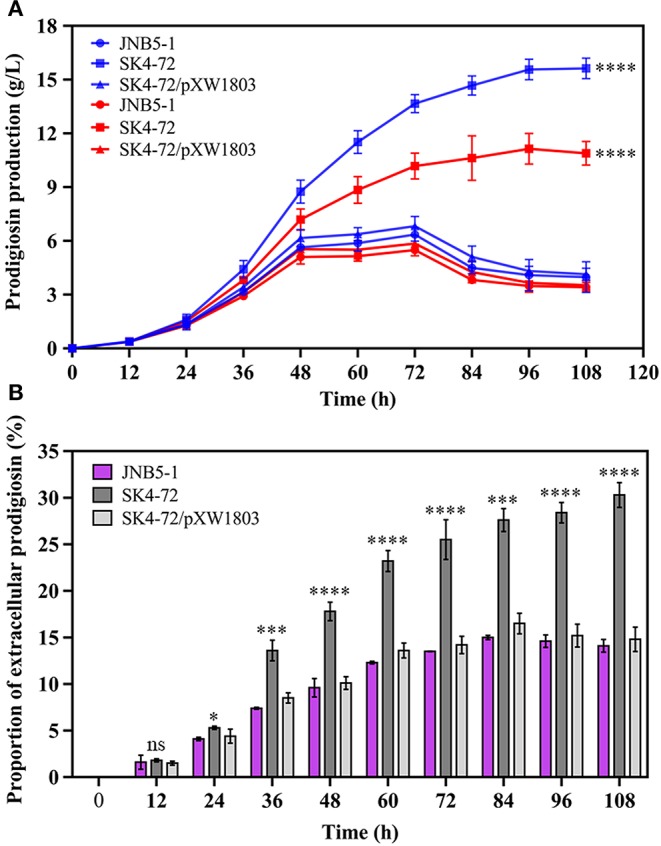
DacA negatively regulates prodigiosin production in fermentation medium. **(A)** Prodigiosin production by JNB5-1, SK4-72, and SK4-72/pXW1803 strains. Blue lines show the total prodigiosin produced by corresponding strains. Red lines show the intracellular prodigiosin concentration of corresponding strains. **(B)** Proportion of extracellular prodigiosin of strains JNB5-1, SK4-72, and SK4-72//pXW1803. SK4-72 is *dacA* disrupted mutant. SK4-72/pXW1803 is *dacA*-complemented strain. For **(A,B)**, the experiments were performed in biological triplicates. Error bars indicate the standard deviations. One-way ANOVA was used to examine the mean differences between the data groups. ns, no significant difference; *p < 0.05; ***p < 0.005; ****p < 0.001.

Further, to analyze the effect of DacA on prodigiosin production in *S. marcescens*, we determined the ability of JNB5-1, SK4-72, and SK4-72/pXW1803 strains to produce prodigiosin in fermentation medium. As shown in Figure 5A, wild-type strain JNB5-1 produced 6.35 g/L of prodigiosin, while the *dacA* mutant SK4-72 yielded 13.66 g/L of prodigiosin after fermentation for 72 h, which was 2.15-times that of wild-type strain, and the highest cumulative amount of 15.63 g/L prodigiosin was obtained after 108 h fermentation, which was 2.46-times that of wild-type strain.

The intracellular prodigiosin concentration of JNB5-1, *dacA* mutant SK4-72, and complementary strain SK4-72/pXW1803 was also determined. Compared with wild-type strain JNB5-1 and complementary strain SK4-72/pXW1803, *dacA* mutant SK4-72 accumulated highest intracellular prodigiosin (Figure 5A, *P*<0.001). Simultaneously, compared with JNB5-1 and SK4-72/pXW1803 strains, the ability of *dacA* mutant SK4-72 to leak prodigiosin into the extracellular environment was significantly enhanced (Figure 5B). These results confirmed that the disruption of *dacA* probably affected cell membrane permeability hence improved the ability of prodigiosin leakage out of the cell membrane, and resulted in alleviated feedback inhibition of prodigiosin on the *pig* gene cluster, ultimately leading to a significant increase in prodigiosin production in the *dacA* mutant SK4-72.

### Effects of D-Ala-D-Ala Carboxypeptidase DacB, DacC, and DacD on Prodigiosin Production

**Figure 6:**
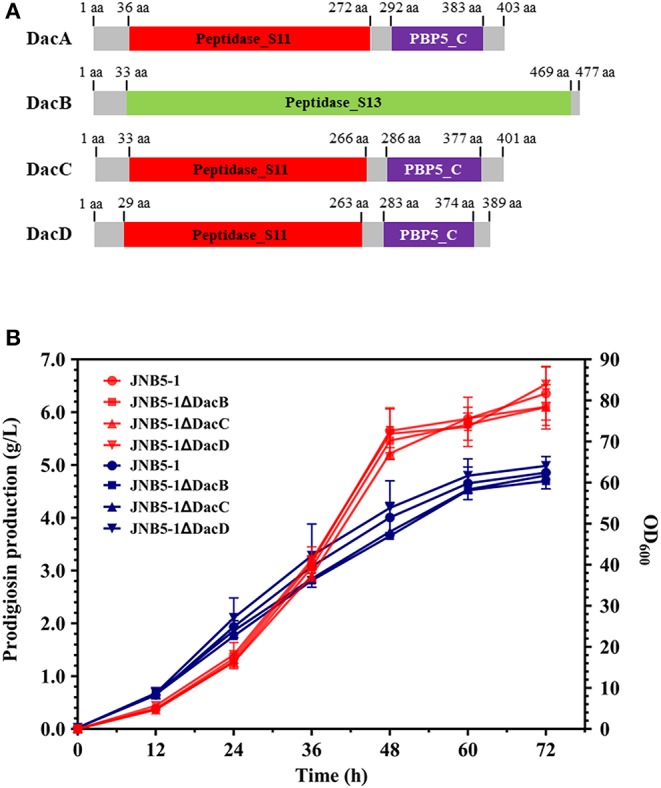
D-Ala-D-Ala carboxypeptidase DacB, DacC, and DacD do not participate in the synthesis of prodigiosin. **(A)** Predicted domain structures of *S. marcescens* D-Ala-D-Ala Carboxypeptidase DacB, DacC, and DacD. **(B)** Effect of *dacB, dacC*, and *dacD* deletion on prodigiosin production and cell growth. Red lines represent the prodigiosin production and blue lines represent biomass (OD_600_). The experiments were performed in biological triplicates. Error bars indicate the standard deviations. JNB5-1 is wild-type strain, JNB5-1ΔDacB is *dacB* mutant. JNB5-1ΔDacC is *dacC* mutant, and JNB5-1ΔDacD is *dacD* mutant.

Four D-Ala-D-Ala carboxypeptidases were encoded in *S. marcescens* UMH8 genome, namely D-Ala-D-Ala carboxypeptidase DacA (BVG90_02415), DacB (BVG90_23445), DacC (BCG90_04675), and DacD (BVG90_09815). Consistent with DacA, D-Ala-D-Ala carboxypeptidase DacB, DacC, and DacD could specifically cleave D-Ala→D-Ala bonds in pentapeptides, resulting in the release of the terminal D-Ala and affecting the synthesis of peptidoglycan (Van Heijenoort, 2011). Further, analyzing the domains of D-Ala-D-Ala carboxypeptidase DacA, DacB, DacC, and DacD of *S. marcescens*, revealed that D-Ala-D-Ala carboxypeptidase DacC and DacD were identical with DacA, having a Peptidase_S11 domain at their N-terminal and a PBP5_C domain at their C-terminal. However, D-Ala-D-Ala carboxypeptidase DacB only has one Peptidase_S13 domain (Figure 6A). Interestingly, although DacA, DacB, DacC and DacD seem to have the same DD-carboxypeptidases activity, their amino acid sequence similarity analysis shows that DacB, DacC, and DacD have low homology with DacA (Figure S3).

To determine whether D-Ala-D-Ala carboxypeptidase DacB, DacC, and DacD also play an important role in regulating the synthesis of prodigiosin in *S. marcescens*, we generated mutants with deletions of DacB, DacC, and DacD-encoding genes, and mutants JNB5-1ΔDacB, JNB5-1ΔDacC, and JNB5-1ΔDacD were obtained (Figure S2). Shake flask fermentation showed that identical with wild-type strain JNB5-1, after 72 h of fermentation, the yield of prodigiosin produced by mutants JNB5-1ΔDacB, JNB5-1ΔDacC, and JNB5-1ΔDacD reached the highest value, of 6.10, 6.11, and 6.53 g/L, respectively. These yields were 95.75, 98.93, and 102.64% that of wild-type strain JNB5-1 (6.35 g/L), respectively, indicating that D-Ala-D-Ala carboxypeptidase DacB, DacC, and DacD were not involved in prodigiosin synthesis in *S. marcescens* (Figure 6B).

## Discussion

Prodigiosin (PG), a red linear tripyrrole pigment (Figure S1) and most prominent member of the prodiginine family, is produced by a number of microorganisms, including *S. marcescens* (Williamson et al., 2006a), *Zooshikella rubidus* (Lee et al., 2011), *Zooshikella ganghwensis* (Lee et al., 2011), *Hahella chejuensis* (Kim et al., 2008a; Krishnamurthy and Belur, 2018), *Streptomyces* sp. (El-Bondkly et al., 2012), and *Serratia nematodiphila* (Gondil et al., 2017; Table 3). Among them, as the most widely studied prodigiosin producing strain, *S. marcescens* has been found to synthesize the highest yield of prodigiosin (49.5 g/L, Table 3). Also, due to important antimicrobial, anticancer and immunosuppressive properties of prodigiosin (Wang et al., 2016; Yip et al., 2019), there is increased research on it and thus optimization of fermentation parameters of *S. marcescens* such as media composition (Chang et al., 2011) and pH (Fender et al., 2012), temperature (Elkenawy et al., 2017), and incubation period (Elkenawy et al., 2017) have been extensively studied for improving prodigiosin production by its natural strains. Simultaneously, many studies have been studied to analyze the metabolic regulatory network of prodigiosin synthesis. Besides the well-studied *pig* gene cluster, a total of 14 genes, associated with prodigiosin synthesis in *S. marcescens*, a few other genes that play important roles in prodigiosin synthesis in *S. marcescens* have also been investigated, including genes *copA* (Williamson et al., 2006b), *crp* (Stella and Shanks, 2014), *hexS* (Stella et al., 2012), *rssB* (Horng et al., 2010), *spnR* (Horng et al., 2002), *eepR* (Shanks et al., 2017), *pigP* (Shanks et al., 2013), *smaI* (Coulthurst et al., 2006), *gumB* (Stella et al., 2018), and *rsbR* (Lee et al., 2017). In this study, *BVG90_02415* (*dacA*) gene encoding D-Ala-D-Ala carboxypeptidase DacA, a negative regulator of prodigiosin production, was screened by constructing the insertion mutant library of Tn*5G* transposon. Compared with wild-type strain JNB5-1, the yield of prodigiosin produced by *dacA* disrupted mutant SK4-72 was 3.89 times (227. 96 mg/L) and 2.46 times (15.63 g/L) higher in LB medium and fermentation medium, respectively (Figures 2A, 5A). The difference in cell growth, cell morphology, *pigA* gene expression level, permeability of inner and outer membranes, and intracellular prodigiosin concentration between JNB5-1 and SK4-72 strains showed that the molecular mechanism for negative regulation of prodigiosin production by DacA probably was that, *dacA* disruption enhanced cell membrane permeability and prodigiosin leakage ability, which in turn alleviated the feedback inhibition of prodigiosin, and improved the expression level of the *pig* operon. Finally, the yield of prodigiosin increased significantly (Figures 2B, 3B–D, 4, 5).

**Table 3:** Microbial production of prodigiosin.

**Strains**	**Medium**	**Prodigiosin production**	**References**
*S. marcescens* BMJ816 and AMJ817	Semi-defined medium [starch, 1.60% (m/v); peptone, 1.07% (m/v); and 1.00% of trace compounds (0.30% CuSO_4_**·**5H_2_O, MgSO_4_**·**7H_2_O, CoSO_4_**·**7H_2_O, FeSO_4_**·**7H_2_O, and MnSO_4_**·**4H_2_O) (m/v)]	103.00 mg/L (shake flask)	Chen et al., 2018
*Serratia marcescens* MO-1	Fermentation medium [yeast extract, 0.40% (m/v); D-Mannitol, 1.00% (m/v); and 0.40% of ram horn peptone (m/v)]	277.74 mg/L (shake flask)	Kurbanoglu et al., 2015
*Serratia marcescens* JX1	Fermentation medium (peptone, 16.00 g/L; glycerol, 20.00 g/L; MgSO_4_, 1.20 g/L; glycine, 2.00 g/L; NaCl 2.00 g/L)	6.50 ± 0.18 g/L (shake flask)	Liu et al., 2013
*Serratia marcescens* TKU011	Fermentation medium [squid pen powder, 1.50% (m/v); MgSO_4_**·**7H_2_O, 0.05% (m/v); K_2_HPO_4_, 0.10% (m/v); FeSO_4_(NH_4_)_2_SO_4_.6H_2_O, 0.10% (m/v)]	2.48 g/L (shake flask)	Liang et al., 2013
*Serratia marcescens* UCP 1549	Fermentation medium (6.00% cassava wastewater, and 2.00% mannitol)	49.50 g/L (shake flask)	De Araujo et al., 2010
*Serratia marcescens* SS-1	Fermentation medium (yeast extract, 5.00 g/L; proline, 10.00 g/L)	2.50 g/L (shake flask)	Wei et al., 2005
*Serratia marcescens* SMΔR	Fermentation medium (yeast extract, 5.00 g/L; proline, 10.10 g/L; 6.0% sunflower oil)	790.00 mg/L (shake flask)	Wei and Chen, 2005
*Serratia marcescens*	Powdered peanut broth	38.75 g/L (shake flask)	Giri et al., 2004
*Serratia nematodiphila* RL2	Nutrient broth medium supplemented with 1.00% lactose and 1.00% yeast extract	0.76 g/L (shake flask)	Gondil et al., 2017
*Streptomyces* sp. NRCF69	Fermentation medium (NaNO_3_, 2.00 g/L; K_2_HPO_4_, 1.00 g/L; KH_2_PO_4_, 1.0 g/L; CaCO_3_, 0.50 g/L; 20.00% of dairy processing wastewater, and 1.00% mannitol)	47.00 g/L (shake flask)	El-Bondkly et al., 2012
*Zooshikella rubidus* S1-1	Marine broth 2216 (MB; Difco)	47.80 mg/L (shake flask)	Lee et al., 2011
*Zooshikella ganghwensis* KCTC 12044^T^	Marine broth 2216 (MB; Difco)	15.40 mg/L (shake flask)	Lee et al., 2011
*Hahella chejuensis* KCTC 2396^T^	Marine broth 2216 (MB; Difco)	28.10 mg/L (shake flask)	Lee et al., 2011
*Hahella chejuensis* KCTC 2396	Fermentation medium (sucrose, 10.00 g/L; peptone, 4.00 g/L; yeast extract, 1.00 g/L; NaCl, 20.00 g/L; Na_2_SO_4_, 9.00 g/L; CaCl_2_, 1.71 g/L; KCl, 0.40 g/L; H_3_BO_3_ 10.00 mg/L; KBr, 50.00 mg/L; NaF, 2.00 mg/L; NaHCO_3_, 45.00 mg/L and Na_2_SiO_3_ 8.10 mg/L)	1.50 g/L (shake flask)	Kim et al., 2008b
*Hahella chejuensis* M3349	Fermentation medium (sucrose, 10.00 g/L; peptone, 8.00 g/L; yeast extract, 2.00 g/L; NaCl, 10.00 g/L; Na_2_SO_4_, 12.00 g/L; CaCl_2_, 1.80 g/L; MgCl_2_, 0.70 g/L; H_3_BO_3_ 22.00 mg/L; Na_2_HPO_4_, 20.00 mg/L and Na_2_SiO_3_ 8.00 mg/L)	2.60 ± 0.18 g/L (shake flask)	Kim et al., 2008a
*Serratia marcescens* JNB5-1	Fermentation medium [Sucrose, 2.00% (m/v); Beef extract, 1.50% (m/v); CaCl_2_, 1.00% (m/v); L-proline, 0.75% (m/v); MgSO_4_.7H_2_O, 0.02% (m/v), and FeSO_4_.7H_2_O, 0.006% (m/v)]	6.35 g/L (shake flask)	This study
*Serratia marcescens* SK4-72	Fermentation medium [Sucrose, 2.00% (m/v); Beef extract, 1.50% (m/v); CaCl_2_, 1.00% (m/v); L-proline, 0.75% (m/v); MgSO_4_.7H_2_O, 0.02% (m/v), and FeSO_4_.7H_2_O, 0.006% (m/v)]	15.63 g/L (shake flask)	This study

DacA, a low-molecular-weight (LMW) penicillin binding protein (PBP), has been extensively studied for its important roles in the synthesis and maintenance of the microorganism cell wall by mediating peptidoglycan crosslinking, structure stabilization, and cell wall modification (Ghosh et al., 2008; Typas et al., 2011). Initially, DacA was considered to be dispensable for *E. coli*, as *dacA* inactivation by mutation does not significantly affect bacterial growth and division (Santos et al., 2002). Subsequent studies have shown that DacA was involved in regulating the cell morphology of *E. coli* as defective for *dacA*, the gene encoding for DacA, exhibit a strong branching phenotype when cell division is blocked (De Pedro et al., 2003). Apart from maintaining cell shape in *E. coli*, DacA was also involved in regulating many other core cellular processes in different microorganism, including maintenances of intrinsic β-lactam resistance in *E. coli* (Sarkar et al., 2010), enhances the accumulation of intracellular soluble peptidoglycan, outer membrane permeability and extracellular protein secretion in *E. coli* (Yang et al., 2018), affects cell growth, cell division and cell shape in pneumococcal (Barendt et al., 2009), influences susceptibility to Lactococcin 972 (Lcn972) in *Lactococcus lactis* (Roces et al., 2012), and requires for *Vibrio cholerae* halotolerance, the *dacA* mutant displays slow growth, aberrant morphology and altered peptidoglycan (PG) homeostasis in LB medium, as well as a profound plating defect (Moll et al., 2015). In this work, we confirmed that D-Ala-D-Ala carboxypeptidase DacA regulated many unknown cellular processes in *S. marcescens*, including cell growth rate (slowed down) (Figure 2B), altered cell morphology, as *dacA* was disrupted, the cell length decreased, and more irregular cells were formed (Figures 3C,D), increased the permeability of inner and outer membranes (Figure 4), and increased the expression level of the *pig* gene cluster, which finally led to the increased prodigiosin production (Figures 1A, 2A, 5A) in *dacA* mutant SK4-72.

Peptidoglycan, the main component of bacterial cell walls, are synthesized and modified by high molecular weight (HMW) PBPs and low molecular weight (LMW) PBPs, which contribute to the firmness and stability of cell structure. The LMW PBPs includes DacA (PBP5), DacB (PBP4), DacC (PBP6), DacD (PBP6b), PBP7, AmpC, and AmpH. According to their different functions, these PBPs can be divided into four categories: (i) monofunctional dd-carboxypeptidase, DacA, DacC, and DacD. (ii) bifunctional dd-carboxypeptidase/dd-endopeptidase, DacB. (iii) monofunctional dd-endopeptidase, PBP7, and (iv) class C β-lactamases, AmpC and AmpH (Nelson and Young, 2001). Among these, dd-carboxypeptidase is the most widely studied, but the understanding of its role *in vivo* is still limited. DacA, DacB, DacC, and DacD can specifically cleave D-Ala→D-Ala bonds in pentapeptides, resulting in the release of the terminal D-Ala and affecting the synthesis of peptidoglycan (Ghosh et al., 2008). Comparing the homology of DacA, DacB, DacC, and DacD encoded in *S. marcescens*, we found that although these proteins all have the carboxypeptidase domain, they displayed less similarities (Figure 6A, Figure S3). Further, analyzing the effects of *dacA, dacB, dacC*, and *dacD* genes on cell growth and prodigiosin synthesis in *S. marcescens*, the results showed that *dacB, dacC*, and *dacD* genes did not affect cell growth and prodigiosin synthesis in *S. marcescens* (Figure 6B). As we mentioned above, disruption of *dacA*, significantly slowed down cell growth, and significantly increased prodigiosin production. These results indicated that there are differences among the LMW PBPs encoded in *S. marcescens*. These differences were also found in other microorganism, including morphological defects accompany the loss of PBP5 but of no other PBP in *E. coli* (Nelson and Young, 2000, 2001); deletion of the *dacA* gene substantially reversed the filamentation phenotype of a temperature-sensitive *ftsK* allele, whereas deletion of *dacB* or *dacC* did not have any significant effect in *E. coli* (Begg et al., 1995); overexpression of DacA caused *E. coli* to become spherical and eventually lyse, DacB, DacC, and DacD are non-lethal (Matsuyama et al., 1992; Vanderlinden et al., 1992; Baquero et al., 1996).

*S. marcescens*, a facultative bacterium belonging to the Enterobacteriaceae family, could produces many useful metabolites and enzymes, such as prodigiosin (Yip et al., 2019), biosurfactant (Matsuyama et al., 1992), oocydin A (Matilla et al., 2017), althiomycin (Gerc et al., 2012), bacteriocins (Foulds, 1972), serratin (Luna et al., 2013), serratiopeptidase (Velez-Gomez et al., 2019), chitinase (Emruzi et al., 2018), and fibrinolytic serine metalloprotease (Krishnamurthy and Belur, 2018), which present enormous potential for application in the pharmaceutical industry. The fact that DacA alleviated the feedback inhibition of prodigiosin by affecting the permeability of cell membrane, indicated that the ability of *dacA* mutant to synthesize other important products probably also improved.

In summary, we described a new regulator DacA which negatively regulates prodigiosin production in *S. marcescens* by enhancing cell membrane permeability and prodigiosin leakage, thereby alleviating feedback inhibition of prodigiosin on the *pig* gene cluster, ultimately enhancing prodigiosin production in *S. marcescens*. More importantly, our work extends the roles of DacA in the control of cell growth, cell morphology and cell membrane permeability. However, further research is needed to reveal the molecular mechanisms how other prodigiosin synthesis-related genes identified in this study regulate prodigiosin production in *S. marcescens*.

## Data Availability Statement

All datasets generated for this study are included in the article/ Supplementary Material.

## Author Contributions

XP performed the experiments and wrote the manuscript. CS carried out measured permeability of intracellular and extracellular membranes of different strains. MT, JZ, JY, and YS constructed and characterized the *dacB, dacC*, and *dacD* deletion mutants. TO and CL helped in writing the manuscript. YZ, MX, and TY contributed to the analysis design and data interpretation. ZR designed the experiments and wrote the manuscript. All authors reviewed the manuscript.

### Conflict of Interest

The authors declare that the research was conducted in the absence of any commercial or financial relationships that could be construed as a potential conflict of interest.
