# Supplementary material for: Loss of Serine-Type D-Ala-D-Ala Carboxypeptidase DacA Enhances Prodigiosin Production in Serratia marcescens
Source: Front Bioeng Biotechnol. 2019 Dec 3;7:367. doi: 10.3389/fbioe.2019.00367 (PMC6901396; doi:10.3389/fbioe.2019.00367)
Supplement: Supplementary file 1 [file Data_Sheet_1.PDF]

Supplementary Material

Xuewei Pan[1], Changhao Sun[1], Mi Tang[1], Chao Liu[1], Jianing Zhang[1], Jiajia You[1], Tolbert Osire[1], Yang Sun[1], You-xi Zhao[2], Meijuan Xu[1], Taowei Yang[1]*, Zhiming Rao[1]*

1. Key Laboratory of Industrial Biotechnology of the Ministry of Education, Laboratory of Applied Microorganisms and Metabolic Engineering, School of Biotechnology, Jiangnan University, Wuxi, 214122, China.

2. Biochemical Engineering College, Beijing Union University, Beijing, 100101, China.

* Corresponding authors: Taowei Yang, E-mail: yangtw@jiangnan.edu.cn; Zhiming Rao, E-mail: raozhm@jiangnan.edu.cn, Tel: 86-510-85916881.

Table S1. Transposon Tn5G insertion sites in prodigiosin synthetic mutants

Strain	Inserted gene (bp)	Insertions site	Protein	Prodigiosin production (mg/L)
SK6-56	*BVG90_02185* (2667)	between 355 bp and 356 bp	phosphoenolpyruvate synthase PigC	0.38±0.07
SK6-11	*BVG90_02185* (2667)	between 355 bp and 356 bp	phosphoenolpyruvate synthase PigC	0.71±0.02
SK2-99	*BVG90_02185* (2667)	between 947 bp and 948 bp	phosphoenolpyruvate synthase PigC	0.93±0.21
ZK2-18	*BVG90_02170* (1017)	between 183 bp and 184 bp	SAM-dependent methyltransferase PigF	0.87±0.15
SK2-85	*BVG90_02170* (1017)	between 183 bp and 184 bp	SAM-dependent methyltransferase PigF	1.14±0.15
SK2-101	*BVG90_21105* (1002)	between 59 bp and 60 bp	transcriptional regulator RbsR	1.46±0.09
ZK2-14	*BVG90_22210* (2553)	between 811 bp and 812 bp	adenylate cyclase	12.41±1.25
SK3-10	*BVG90_22210* (2553)	between 811 bp and 812 bp	adenylate cyclase	11.57±1.23
SK5-41	*BVG90_22210* (2553)	between 811 bp and 812 bp	adenylate cyclase	13.14±0.68
SK3-50	*BVG90_00600* (1803)	between 50 bp and 51 bp	sulfite reductase [NADPH] flavoprotein, alpha-component	9.42±1.33
ZK2-27	*BVG90_00600* (1803)	between 42 bp and 43 bp	sulfite reductase [NADPH] flavoprotein, alpha-component	12.56±1.10
SK4-51	*BVG90_17190* (525)	between 48 bp and 49 bp	cupin	3.12±0.49
SK3-47	*BVG90_17190* (525)	between 56 bp and 57 bp	cupin	4.14±0.86
ZK2-29	Intergenic region (630)	between 199 bp and 200 bp	downstream genes are *BVG90_24610* and *BVG90_24615*	9.70±1.02
SK2-11	Intergenic region (630)	between 199 bp and 200 bp	downstream genes are *BVG90_24610* and *BVG90_24615*	11.35±0.78
SK3-24	Intergenic region (630)	between 199 bp and 200 bp	downstream genes are *BVG90_24610* and *BVG90_24615*	7.49±0.63
SK6-61	Intergenic region (238)	between 6 bp and 7 bp	downstream gene is *BVG90_18410*	3.51±0.21
SK6-16	*BVG90_14345* (327)	between 178 bp and 179 bp	phage holin, lambda family	5.79±0.27
SK6-71	*BVG90_02010* (684)	between 393 bp and 394 bp	DNA-binding transcriptional repressor AcrR	6.10±0.56
SK6-69	Intergenic region (856)	between 48 bp and 49 bp	downstream genes are *BVG90_13460* and *BVG90_13465*	6.79±1.54
SK6-60	*BVG90_17590* (483)	between 357 bp and 358 bp	hypothetical protein	7.09±1.50
SK6-46	*BVG90_22540* (1632)	between 672 bp and 673 bp	ubiquinone biosynthesis regulatory protein kinase UbiB	7.94±0.37

ZK2-31	*BVG90_00235* (294)	between 35 bp and 36 bp	acetolactate synthase isozyme 1 small subunit	8.22±0.53
SK6-37	*BVG90_04215* (1428)	between 407 bp and 408 bp	alkaline phosphatase	11.35±1.46
SK6-32	*BVG90_22885* (3096)	between 2386 bp and 2387 bp	peptide synthetase	11.79±0.25
SK6-47	*BVG90_03255* (894)	between 744 bp and 745 bp	LysR family transcriptional regulator	12.16±1.27
SK6-84	*BVG90_02925* (1191)	between 103 bp and 104 bp	hypothetical protein	11.59±1.08
SK3-13	*BVG90_07405* (1338)	between 206 bp and 207 bp	alcohol dehydrogenase	15.81±1.30
ZK66	*BVG90_22495* (945)	between 483 bp and 484 bp	LysR family transcriptional regulator MetR	46.88±2.24
SK4-72	*BVG90_02415* (1212)	between 555 bp and 556 bp	D-Ala-D-Ala carboxypeptidase DacA	57.69±1.84
SK3-6	*BVG90_00775* (186)	between 9 bp and 10 bp	carbon storage regulator RsmA	63.11±1.99
SK3-15	*BVG90_13460* (945)	between 112 bp and 113 bp	transcriptional regulator LrhA	130.14±2.60

Figure. S1

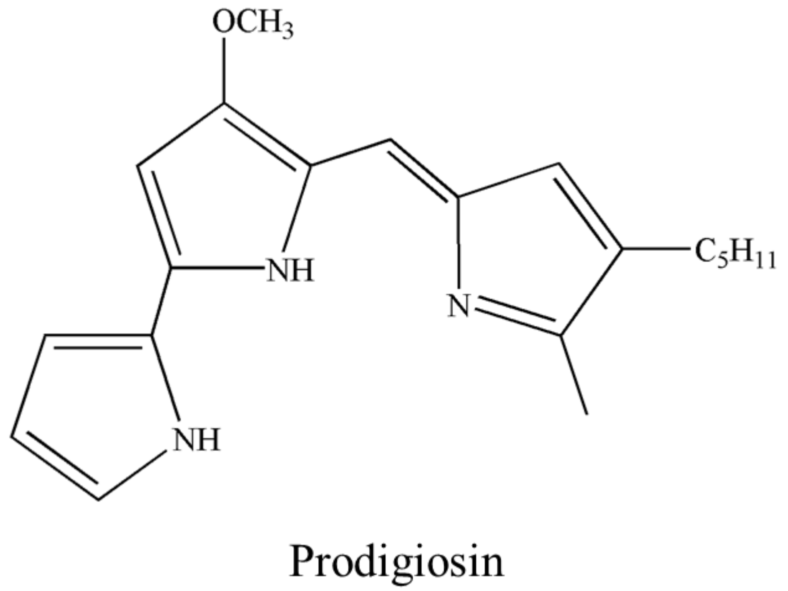

Figure. S1. Chemical structure of prodigiosin.

Figure. S2

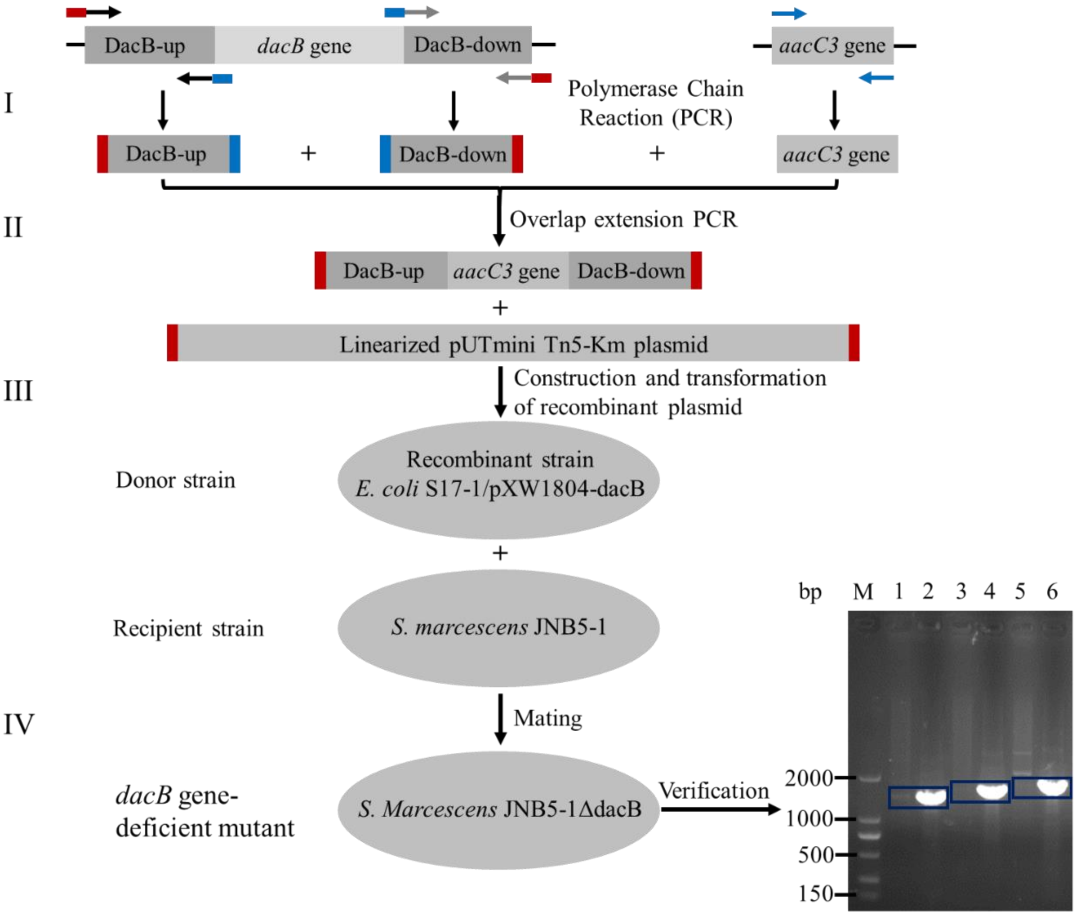

Figure. S2. Flow chart for screening *dacB* mutant JNB5-1ΔDacB, *dacC* mutant JNB5-1ΔDacC, and *dacD* mutant JNB5-1ΔDacD. DacB-up and DacB-down represent the upstream and downstream homologous arms of the *dacB* gene, respectively, with a length of about 1000 bp. Red rectangle represents homologous sequence with pUTKm plasmid, and blue rectangle represents homologous sequence with *aacC3* gene. I, II, III and IV represent the four steps in the construction of *dacB* mutant JNB5-1ΔDacB, respectively. M: DNA Marker (2000 bp), 1-6 was used to demonstrate the deletion of *dacB*, *dacC* and *dacD* genes in mutants JNB5-1ΔDacB, JNB5-1ΔDacC and JNB5-1ΔDacD by PCR. For PCR analysis, the forward primers complemented the upstream homologous arm sequences of *dacB*, *dacC* and *dacD* genes, while the reverse primers complemented the apramycin resistance genes. 1-2: Using control JNB5-1 and *dacC* mutant JNB5-1ΔDacC genome as templates, PCR analysis to demonstrate whether the *dacC* gene was successfully knocked out; 3-4: Using control JNB5-1 and *dacB* mutant JNB5-1ΔDacB genome as templates, PCR analysis to demonstrate whether the *dacB* gene was knocked out successfully; 5-6: Using control JNB5-1 and *dacD* mutant JNB5-1ΔDacD genome as templates, PCR analysis to demonstrate whether the *dacD* gene was knocked out successfully.

Figure. S3

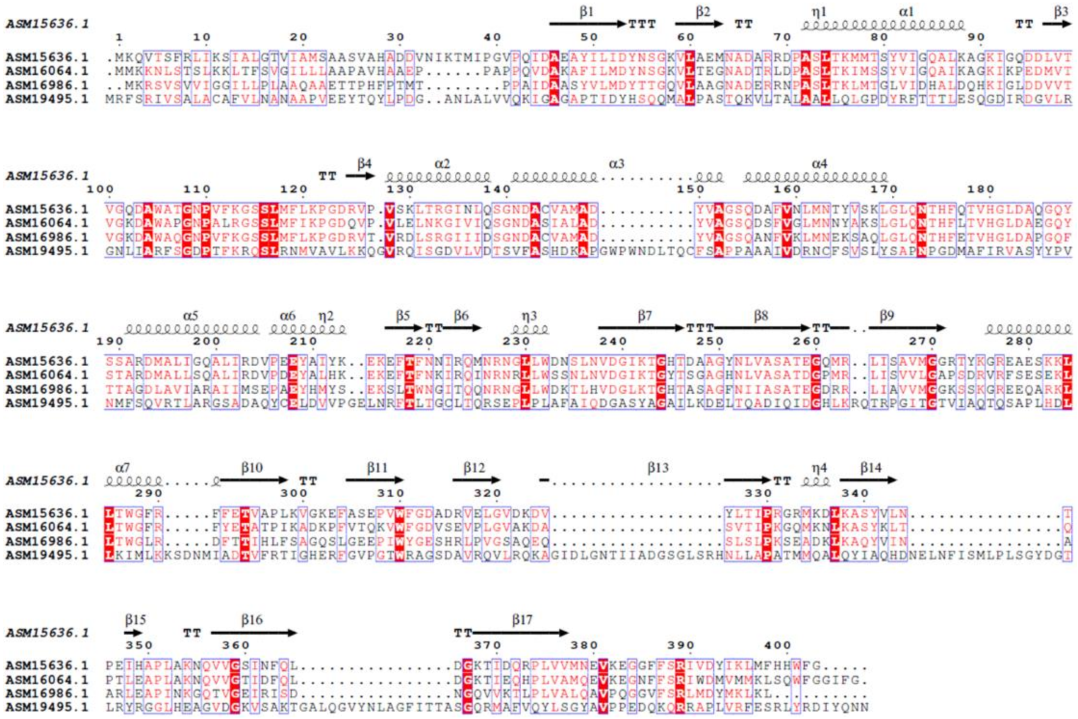

Figure. S3. Homology analysis of the protein sequences of D-Ala-D-Ala carboxypeptidase DacA, DacB, DacC and DacD encoded in *S. marcescens*. ASM15636.1 indicates D-Ala-D-Ala carboxypeptidase DacA, ASM16064.1 indicates D-Ala-D-Ala carboxypeptidase DacC, ASM16986.1 indicates D-Ala-D-Ala carboxypeptidase DacD, and ASM19495.1 indicates D-Ala-D-Ala carboxypeptidase DacB, respectively.
